# Mitochondrial Dysfunction, Macrophage, and Microglia in Brain Cancer

**DOI:** 10.3389/fcell.2020.620788

**Published:** 2021-01-15

**Authors:** Rongze Olivia Lu, Winson S. Ho

**Affiliations:** ^1^Department of Neurosurgery, Dell Medical School, University of Texas at Austin, Austin, TX, United States; ^2^Mulva Clinic for the Neurosciences, Dell Medical School, University of Texas at Austin, Austin, TX, United States

**Keywords:** tumor associated macrophages and microglia, mitochondrial dysfunction, mitochondrial DNA, brain cancer, inflammatory response

## Abstract

Glioblastoma (GBM) is the most common malignant brain cancer. Increasing evidence suggests that mitochondrial dysfunction plays a key role in GBM progression as mitochondria is essential in regulating cell metabolism, oxidative stress, and cell death. Meanwhile, the immune microenvironment in GBM is predominated by tumor-associated macrophages and microglia (TAM), which is a heterogenous population of myeloid cells that, in general, create an immunosuppressive milieu to support tumor growth. However, subsets of TAMs can be pro-inflammatory and thereby antitumor. Therapeutic strategies targeting TAMs are increasingly explored as novel treatment strategies for GBM. The connection between mitochondrial dysfunction and TAMs phenotype in the tumor microenvironment is unclear. This review aims to provide perspectives and discuss possible molecular mechanisms mediating the interplay between glioma mitochondrial dysfunction and TAMs phenotype in shaping tumor immune microenvironment.

## Introduction

Brain cancer is the leading cause of cancer-related deaths in patients younger than 35 (Wen and Kesari, 2008). Glioblastoma (GBM) accounts for 70% of malignant primary brain tumors, taking more than 13,000 lives in the United States each year (Wen and Kesari, 2008). GBM remains an incurable cancer with a 5 year survival <5% and median survival <15 months (Arrigo et al., 2012; Koshy et al., 2012). The current standard of care is a combination of surgery, chemotherapy, and radiation, which are of limited efficacy and often cause devastating neurological side effects. Therefore, safer and more effective therapeutic modalities are urgently needed for GBM.

GBM tumor microenvironment is predominated by a heterogenous population of myeloid cells composed of brain-resident microglia and bone-marrow-derived macrophages, which are collectively referred to as tumor-associated macrophages and microglia (TAMs). TAMs represent about 40% of tumor mass in GBM (Kennedy et al., 2013). Tumor cells has been shown to dynamically interact with TAMs, a phenotypically plastic population, to induce an immunosuppressive microenvironment that facilitates tumor growth and evasion of immunosurveillance. Meanwhile, mitochondrial dysfunction is a hallmark of GBM. Current studies mostly focus on the impact of mitochondrial dysfunction on intrinsic tumor function. How tumor mitochondrial dysfunction influences the function of non-tumor cells such as TAMs is not well-studied. In this review, we will discuss the key features of mitochondrial dysfunction in glioma and provide our perspective on how GBM mitochondrial dysfunction can regulate immune response in the tumor microenvironment.

## Heterogenous Macrophages and Microglia in Glioma

The prevalence of TAMs drives the immunosuppressive tumor microenvironment in GBM. They are associated with poor prognosis in GBM patients. Interestingly, depletion of TAMs failed to demonstrate any clinical benefit (Butowski et al., 2016), which suggests that a subset of TAMs is necessary for effective antitumor immunity. In classic *in vitro* macrophage polarization experiments, macrophages can be polarized into M1-like (antitumor) macrophage, which produces pro-inflammatory cytokines [interleukin (IL)-12, tumor necrosis factor alpha (TNF-α), IL-1ß] or M2-like (protumor) macrophage, which produces immunosuppressive cytokines such as transforming growth factor beta (TGF-β) and IL-10. However, recent evidence suggests that this M1 and M2 classification is an oversimplification *in vivo*. Single cell sequencing of TAMs in human GBM samples showed a highly heterogenous population that frequently coexpress pro-inflammatory (M1) cytokines, such as TNF-α, IL1ß, and immunosuppressive (M2) cytokines, such as IL10, Arg1, vascular endothelial growth factors (VEGFs), in individual cells at the transcription level (Müller et al., 2017; Takenaka et al., 2019). Flow cytometry of patient samples also confirm coexpression of both M1 marker CD86 and M2 marker CD206 on the protein level (Müller et al., 2017). *In vitro*, glioma-conditioned medium also induced upregulation of both M1-like (*Stat1, Cd274, Il1b, Tnfa*, and *Il27*) and M2-like (*Arg1, Vegfa, Il10, Klf4*, and *Pparg*) markers in macrophages, consistent with TAMs phenotype in patient samples (Takenaka et al., 2019).

In addition to their functionally heterogenous phenotypes, TAMs have two distinct cells of origin. Blood-derived macrophages originate peripherally from bone barrow monocytes precursors. These cells normally are excluded from the brain with an intact blood–brain barrier (BBB). In glioma, the BBB is partially compromised, allowing for peripheral monocytes to infiltrate into tumors and differentiate into macrophages. This process is dependent on enhanced expression of the monocyte chemoattractant family of proteins (MCPs) from tumors and their receptors on monocytes (such as CX3CR1 and CCR2). Peripheral monocytes are CX3CR1^Lo^CCR2^Hi^, but once they are recruited to the tumor, they transition into CX3CR1^Hi^CCR2^Lo^ macrophage or CX3CR1^Hi^CCR2^−^ cells (Chen et al., 2017). Brain-resident microglia is the other major cell type of TAMs. Microglia are unique resident macrophages that are essential for normal brain function. Fate-mapping and lineage-tracing studies have shown that immature yolk sac progenitor cells are the predominant source of brain microglia.

Recent studies of TAMs have demonstrated significant spatial and functional heterogeneity within these two major subsets of TAMs. Single-cell RNA sequencing (scRNAseq) showed that blood-derived macrophages are enriched in perivascular and necrotic regions, with upregulations of immunosuppressive genes and altered metabolic gene signatures (Müller et al., 2017). Importantly, infiltration of blood-derived macrophages instead of microglia correlates with poor survival in low-grade glioma (Müller et al., 2017). ScRNAseq of mouse glioma shows that there are eight to nine clusters of microglia and three clusters of blood-derived macrophages (Ochocka et al., 2019). Among the microglia subsets, there are three major subgroups: one with high expression of homeostatic microglia signature genes, one with high transcriptional activity of gene that inhibit nuclear factor kappa B (NFκB) signaling, and one with increased expression of genes for antigen presentation (Ochocka et al., 2019). For blood-derived macrophages, three clusters were also identified: one characterized by an inflammatory monocyte signature (Ly6c2, Ccr2, Tgfbi), one with intermediate state of mixed monocytes and macrophage signature (Ly6c2, Tgfbi), and one with differentiated macrophage signature (Ly6c2, Ifitm2, Ifitm3, S100a6) (Ochocka et al., 2019). These findings demonstrate the dynamic plasticity of both microglia and macrophages in the tumor microenvironment.

Recently, another type of tissue-resident macrophage was identified: border-associated macrophages (BAMs) with tissue-specific transcriptional signatures that reside in the dura mater, subdural meninges, and choroid plexus (Mrdjen et al., 2018; Van Hove et al., 2019). BAM is also heterogenous, which can be characterized into subsets based on CD38, major histocompatibility complex II (MHCII) expression, and their specific location in the central nervous system (CNS) compartment (Mrdjen et al., 2018; Van Hove et al., 2019). In addition, transcriptional factor IRF8 has been identified to regulate maturation and diversity of BAM (Van Hove et al., 2019).

Given the vast heterogeneity of TAMs, it is critical that any novel strategies targeting TAMs need to take into account the effect on specific TAMs subpopulations and the impact on cancer immunity.

## Innate Immune Response in TAMs and Cross Talk with Glioma Cells

Macrophages and microglia are critical innate immune cells that sense signals through pattern recognition receptors (PRRs) such as Toll-like receptors (TLRs). In the tumor microenvironment, TAMs are highly plastic and can be educated by cancer cells. To identify molecular mechanisms regulating immune response mediated by TAMs, it is critical to develop TAMs-based clinical strategies. A previous study has shown that, although TAMs in glioma express TLRs and surface MHC-II, they are not sufficient to mediate inflammatory/antitumor response when stimulated with TLR agonist (Hussain et al., 2006). In addition, TAMs are unable to activate CD4 T cells *in vitro* culture (Hussain et al., 2006). TLR2 along with TLR1 or TLR6 on tumor-associated microglia mediates an immunosuppressive role by enhancing production of matrix metalloprotease (MMP) to facilitate tumor invasion (Vinnakota et al., 2013; Hu et al., 2014). NFκB signaling pathway plays a significant role in macrophages and microglia-mediated inflammatory response. From transcriptional gene analysis, it has been shown that TLR signaling pathway genes was reduced in high-grade gliomas compared to low-grade gliomas. In particular, IKKβ, a key protein leading to NFκB activation, is downregulated in high-grade glioma, and downregulation of IKKβ is correlated with immunosuppressive gene signatures in tumors (Mieczkowski et al., 2015). A recent study support these findings by identifying a subset of microglia that highly express genes that inhibiting NFκB signaling (Ochocka et al., 2019).

Type I interferon (IFN) plays a critical role in antitumor immunity by promoting antigen presentation in dendritic cells, enhancing CD8 T cell proliferation, and inhibiting regulatory T cells (Fujita et al., 2010; Ohkuri et al., 2014). Loss of type I IFN signaling has been linked to tumorigenesis in glioma. Intratumoral administration of stimulator of interferon gene (STING) agonist to enhance type I IFN production improved mouse survival in glioma models. Mechanistically, type I IFN suppresses FOXP3 regulatory T cells and therefore increases IFNγ-producing CD8 T cells (Ohkuri et al., 2014). In addition, type I IFN has an inhibitory effect on proliferation of glioma stem cell (GSCs) and inhibits GSC stemness (Du et al., 2017).

Previous studies suggest that DCs are the major type I IFN-producing cells by sensing dying tumor cells (Deng et al., 2014). However, in glioma, CD11b^+^ myeloid cells express higher transcriptional level of type I IFN than CD11c^+^ DCs (Ohkuri et al., 2014). In addition, more recent studies demonstrated macrophage phagocytes double-stranded DNA (dsDNA) from dying tumor cells to elicit downstream STING-type I IFN signaling pathways, resulting in strong tumor immunogenicity (Ahn et al., 2018; Zhou et al., 2020b). Considering the prevalence of TAMs in glioma, we speculate that TAMs play a critical role in sensing dying tumor cells and thereby producing type I IFN, particularly in the context of radiation-treated glioma. This is an area worth investigating more efforts to delineate the cross talk between dying tumor cells and TAMs response. Furthermore, few studies have demonstrated the role of cyclic guanosine monophosphate–adenosine monophosphate synthase (cGAS)–STING pathway in microglia. In stroke model, cGAS–STING signaling polarized microglia into pro-inflammatory phenotypes by increasing TNFα production (Jiang et al., 2020). A neuroinflammation model showed activation of STING-dependent type I IFN in microglia reduced microglia activity and attenuate neuroinflammation (Mathur et al., 2017). These studies suggest that the role of cGAS–STING type I IFN signaling in microglia is highly dependent on the disease models, and it is highly interesting to investigate the role of this pathway in microglia-mediated antitumor immunity.

**Figure 1:**
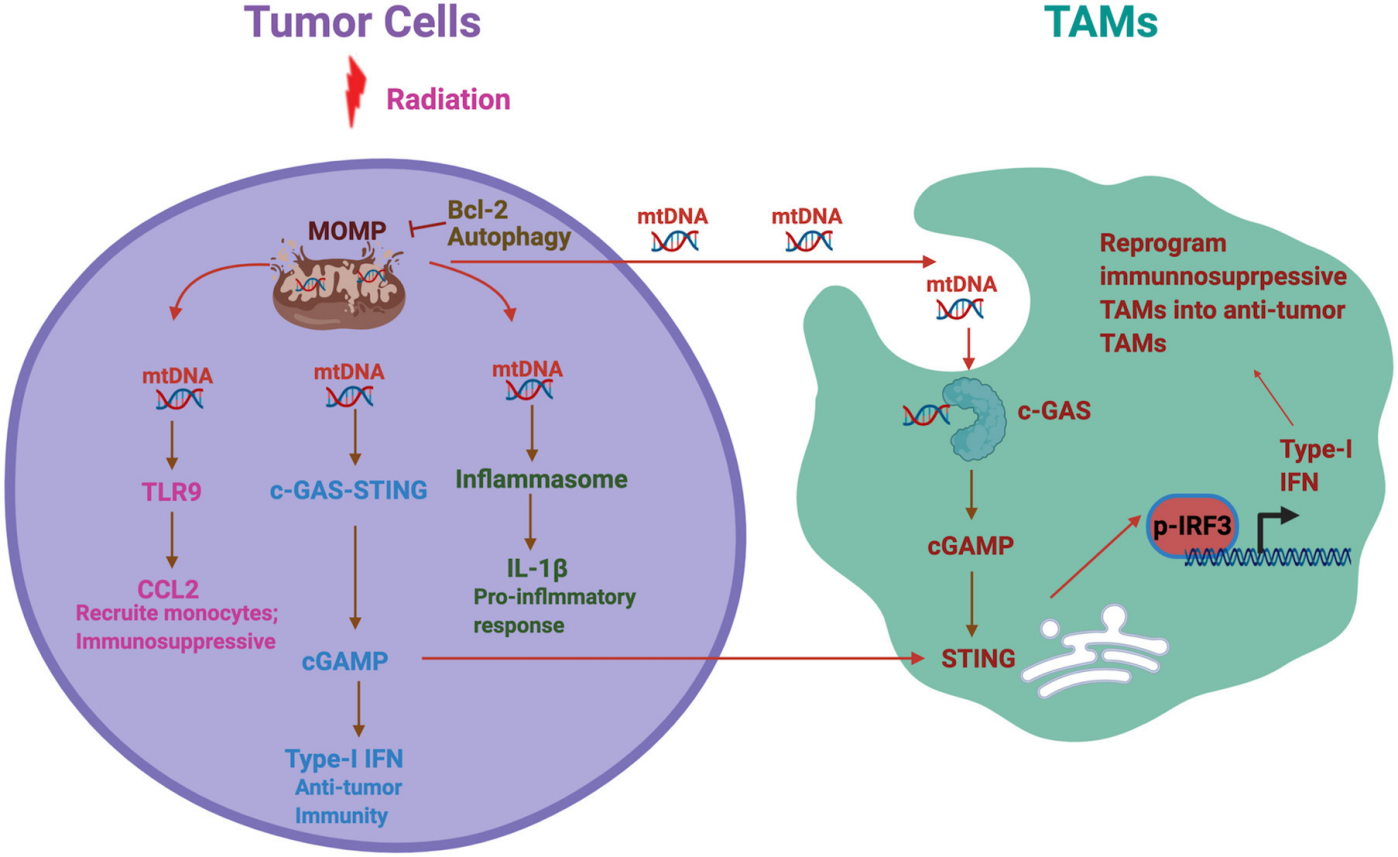
Mitochondrial DNA (mtDNA)-induced inflammatory response in cancer. A schematic summary of the potential cross talk between tumor mitochondrial dysfunction, hence mtDNA release, and tumor-associated macrophages and microglia (TAMs) functional phenotype. Radiation and other mitochondrial stress induce mitochondrial outer membrane permeabilization (MOMP) and thereby mtDNA release to the cytosol. Antiapoptotic factor Bcl-2 and autophagy inhibit the MOMP and prevent mtDNA release. mtDNA further activates multiple inflammatory signaling pathways including Toll-like receptor 9 (TLR9), stimulator of interferon gene (STING), and inflammasome, each of which plays different roles in immune response. On the other side, we hypothesize that tumor-derived mtDNA also stimulates TAMs to activate cGAS–STING pathways, resulting in type-I interferon (IFN) production in TAMs. In addition, tumor derived cGAMP can be secreted as an immunotrasmitter that enter TAMs to stimulate STING and thereby Type 1 IFN production. This process could reprogram the TAMs in the tumor microenvironment from immunosuppressive to antitumor phenotypes.

In summary, innate immune response of TAMs is educated by tumor cells and significantly different from other inflammatory models. Therefore, to identify cancer intrinsic factors that influence the change of TAMs phenotypes in mediating immune response could be critical for understanding the cross talk between cancer cells and TAMs and thereby the development of TAMs-based cancer treatment (Figure 1). Mitochondrial dysfunction is a hallmark of glioma and impact multiple functions of glioma. How mitochondrial dysfunction in tumor influences innate immune responses is unclear in glioma. We will provide several perspectives and hypothesis based on previous findings.

## Mitochondrial Dysfunctions in Glioma

Mitochondria play a significant role in the number of essential cellular processes including metabolism, management of oxidative stress, and apoptosis. The catabolic engine of mitochondria produces redox reactions to create flux of electrons across the inner mitochondrial membrane to produce ATP. In addition, mitochondria also mediate programmed cell death, which is controlled by mitochondrial outer membrane permeabilization (MOMP) (Lopez and Tait, 2015). Following MOMP, mitochondrial inner space protein cytochrome c is released into cytosol to activate caspases, resulting in apoptotic cell death (Lopez and Tait, 2015). Furthermore, mitochondria are also a main source of reactive oxygen species (ROS), which is produced by the electron transport chain of the inner mitochondrial membrane. ROS could cause oxidative damage to mitochondrial and genomic DNA, affecting mitochondrial metabolic ability to generate ATP and widely impact many cellular functions (Murphy, 2009).

Mitochondrial dysfunction is a hallmark of cancer (Pavlova and Thompson, 2016; Vander Heiden and Deberardinis, 2017). In glioma, mitochondrial function is impaired due to significant alteration in mitochondrial genome, leading to altered morphology and abnormal bioenergetics including enhanced generation of ROS (Guntuku et al., 2016; Strickland and Stoll, 2017). This reprograming causes a shift in metabolism in glioma cells, creating a decoupling event in the metabolic pathway, leading to enhanced utilization of glycolytic metabolism instead of oxidative phosphorylation pathway (Guntuku et al., 2016; Strickland and Stoll, 2017). As a result of this switch to glycolytic metabolism, known as the Warburg effect, abnormal mitochondria phenotypes arise (Guntuku et al., 2016; Strickland and Stoll, 2017). These abnormal mitochondria phenotypes include swelling and osmophilic granules, which is thought to play a role in the invasive nature or pathobiology of gliomas (Guntuku et al., 2016; Strickland and Stoll, 2017).

Elevated mtROS from dysfunctional mitochondria induces oxidative stress, which lead to apoptosis through activation of p53 and downstream of bcl-2 family protein. However, glioma cells can survive this oxidative stress through multiple mechanisms. Oncogenic activation of PI3K/AKT pathway in glioma can induce p53 degradation to avoid mitochondrial-stress-induced apoptosis. Consistently, glioma cells with p53 mutations are also resistant to mtROS-induced apoptosis (Guntuku et al., 2016). In addition, ROS generation facilitates protumorigenesis transcription factors HIF1a and nuclear factor erythroid 2-related factor 2 (NRF2) inevitably driving proliferation and promotion of cell viability (Pavlova and Thompson, 2016). In summary, mitochondrial dysfunction in glioma regulates numerous cancer intrinsic pathways involving tumor metabolism, survival, proliferation, and cell death. In glioma tumor microenvironment, glioma cells dynamically interact with other cells such as macrophage/microglia, astrocytes, and neurons (Antunes et al., 2020). How mitochondrial dysfunction in glioma affects the cross talk between glioma cells and other cells are not clear. Below, we will discuss the possible link between mitochondrial dysfunction in glioma with TAMs-mediated cancer immunity.

## Mitochondrial Dysfunction and Immune Response

Mitochondria evolutionarily originate from an endocytic event of a proteobacteria (Zhang et al., 2010; McArthur et al., 2018). Therefore, mitochondrial DNA (mtDNA), similar to bacteria DNA, has damage-associated molecular patterns (DAMPs), which are conserved motifs that potently bind PRRs expressed by innate immune cells such as TAMs. The release of mtDNA to cytosol or extracellular environment is a process tightly controlled by cells and can potently activate innate immune cells. Mitochondrial dysfunctions and pathological leakage of mtDNA has been associated with many diseases such as infections, inflammatory diseases, or stress induced by irradiation or trauma (Patrushev et al., 2004, 2006; García et al., 2005; Zhang et al., 2010; Shimada et al., 2012; West et al., 2015).

Mitochondrial-mediated apoptosis is an immunologically silent event that does not trigger downstream innate immunity, as the presence of apoptotic caspases suppresses cGAS–STING-mediated type I IFN production (Rongvaux et al., 2014; White et al., 2014). However, BAK/BAX-mediated apoptosis is able to trigger mtDNA-dependent type I production by forming pores on mitochondrial outer membrane, allowing the release of mtDNA (McArthur et al., 2018). In addition to activating STING–type I IFN pathway, mtDNA induces IL-1ß production during apoptosis by activating NLRP3 inflammasome (Shimada et al., 2012).

In cancer models, emerging evidence suggest that mtDNA plays a critical role in antitumor immunity, particularly in the setting of radiation therapy. mtDNA from tumor receiving radiation or anti-CD47 antibody, which blocks the “do not eat me” signal, promotes dendritic cells' ability to cross present antigen to CD8 T cells (Xu et al., 2017; Fang et al., 2020). Mechanistically, radiation induces MOMP and release of mtDNA into the cytosol, thereby potently activating type I IFN production, which is required for effective abscopal response to radiation therapy (Yamazaki et al., 2020). Evidence using high-resolution confocal and conventional microscopy demonstrates that, following radiation of tumors, cytosolic dsDNA colocalizes with mitochondrial elements rather than nuclear envelope markers. This suggests that mtDNA rather than nuclear DNA is the primary driver of type I IFN production, highlighting the importance and potency of mtDNA in mediating RT-induced innate immune response (McArthur et al., 2018; Yamazaki et al., 2020). The production of type I IFN is severely compromised in the presence of autophagy and the antiapoptotic protein Bcl2, both of which inhibit MOMP and its immunological response (Yamazaki and Galluzzi, 2020; Yamazaki et al., 2020). Therefore, a potential strategy for brain tumor treatment is to combine radiation therapy with Bcl2 or autophagy inhibitor in order to achieve long-lasting immune-mediated antitumor immunity and abscopal effect. On the other hand, there is evidence that mtDNA in certain context could also promote immunosuppression in the tumor microenvironment. mtDNA has been reported to activate TLR9 in hepatocellular carcinoma to induce CCL2 production, which in turns promotes macrophage infiltration and sustain the immunosuppressive phenotype of macrophages (Bao et al., 2019). In this context, the release of mtDNA to cytosol is facilitated by hypoxia tumor microenvironment (Liu et al., 2015).

In glioma, the connection between tumor-derived mtDNA and the immune response is not clear. As we discussed above, mitochondrial dysfunction is the hallmark of glioma and has been associated with release of mtDNA to cytosol and extracellular space in many diseases' models. Particularly, hypoxia, which is also a hallmark of glioma, has been shown to facilitate the release of mtDNA to cytosol. Therefore, we postulate that the release of tumor-derived mtDNA to cytosol occurs commonly in glioma. Considering the abundance of TAMs in the glioma microenvironment and the mtDNA-dependent inflammatory response TAMs demonstrate in other diseases models and cancer types (Collins et al., 2004; Bao et al., 2019), we hypothesize that mtDNA is an important driver of innate immunity in glioma. Importantly, considering radiation is the standard of care in GBM, we also posit that that tumor-derived mtDNA is critical in mediating response to radiation therapy through STING–type I IFN pathway. Previous findings of mtDNA-mediated type I IFN production is focused on cancer intrinsic signaling (Yamazaki et al., 2020); it is therefore critical in future studies to investigate the effect of tumor-derived mtDNA on TAMs.

The source of cGAS-mediated cyclic guanosine monophosphate–adenosine monophosphate (cGAMP) generation in the tumor microenvironment is not completely elucidated. Some evidence suggest that tumor cells are phagocytosed by DCs or TAMs; then, tumor DNA (nuclear or mtDNA) subsequently enter the cytoplasm from the phagosomes to activate the cGAS–STING axis. Several recent studies, however, demonstrated that type1 IFN production is dependent on tumor cGAS to generate cGAMP, which can then be secreted as an immunotrasmitter that enter other cells such as DC/TAMs to stimulate STING and thereby type 1 IFN production (Marcus et al., 2018; Carozza et al., 2020; Zhou et al., 2020a). This mechanism is supported by recent discoveries of multiple cGAMP transporters such as SLC19A1 and LRRC8 (Luteijn et al., 2019; Zhou et al., 2020a). In addition, a cGAMP hydrolase, ENPP1, was identified as a tumor-expressed surface and secreted enzyme to clear extracellular cGAMP as a means of preventing activation of innate immune cells. ENPP1 inhibitor could synergize with radiation to enhance immune-mediated tumor rejection by increasing cGAMP availability in the tumor microenvironment (Carozza et al., 2020). Therefore, it goes to reason that a rational strategy in glioma treatment is to (1) enhance production of cytosolic mtDNA in order to increase tumor-derived cGAMP and (2) to maximize availability of cGAMP in the tumor microenvironment. Increasing mtDNA damage in glioma to enhance its immunogenicity would be a novel approach to treat GBM. A recent study showed that monoamine oxidase B (MAOB) is highly expressed in GBM mitochondria, and targeting MAOB resulted in mitochondrial-specific DNA damage and efficacy against GBM in mouse xenograft models (Sharpe et al., 2015). Whether this approach can augment cGAMP–STING activation and thereby tumor immunogenicity has not been explored. It is also important to note that, given the heterogeneity of TAMs, which subset of TAMs has the greatest sensitivity to cGAMP or has the greatest capacity to produce type I IFN has not been well-studied. Any treatment strategies to enhance TAMs sensitivity to cGAMP could therefore also be beneficial. Recently, blockade of a phagocytic receptor, MerTK, in macrophage has been shown to enhance cGAMP uptake by enhancing opening of an ATP-gated channel, P2X7R, that mediate cGAMP uptake (Zhou et al., 2020b). Whether merTK is expressed differentially in different subsets of TAMs is not clear. In conclusion, further study of the relationship between mitochondrial dysfunction and antitumor immune response could uncover novel immunotherapeutic strategies against GBM.

## Lessons from Neurodegenerative Disease

It is well-recognized that inflammation plays a critical role in the pathogenesis of neurodegenerative disorder such as Parkinson's (PD) and Huntington's (HD) disease. Mitochondrial dysfunction is a common feature, and the molecular mechanisms are increasingly being elucidated. For example, mutations in Pink1 or Prkn, which function within the same biochemical pathway, were identified in familial PDs (Kitada et al., 1998; Valente et al., 2004). It is essential for mitochondrial quality control and is responsible for removing damaged mitochondria through mitophagy (Pickrell and Youle, 2015). A recent study showed that in Pink1−/− or Prkn−/− mice, acute-exercise-induced or chronic mtDNA mutation-induced mitochondrial stress leads to STING-mediated type I IFN response resulting in systemic inflammation. This effect was completely abolished in the absence of STING in STING^gt/gt^ mice crossed with Pink1−/− or Prkn−/− mice. Pink1/Prkn deletion in *mutator* mice with a proofreading-defective mtDNA polymerase showed enhanced circulating mtDNA and higher mtDNA to nuclear DNA ratio (Sliter et al., 2018). While chronic inflammation may contribute to pathogenesis of PDs, in the setting of the immunosuppressive tumor microenvironment, Pink1/Prkn deficiency may be beneficial to enhance STING-mediated type I IFN production especially in the setting of mitochondrial stress such as radiation treatment. Targeting Pink1/Prkn pathway may therefore be a novel strategy against GBM. Another potential insight could be drawn from HD. Mutations of the HD causative gene huntingtin result in impaired mitochondrial protein import and thereby increase in mitochondrial oxidative stress resulting in enhanced mtDNA damage (Yano et al., 2014). Using a mouse model of HD, a study showed that deficiency in melatonin, a potent free radical scavenger, leads to increased cytosolic mtDNA release and potent activation of the cGAS/STING/IRF3 pathway, resulting in a pathological inflammatory response causing synaptic loss and neurodegeneration (Jauhari et al., 2020). Similar to PINK1/PRKN deficiency in PD, while defect in melatonin may be detrimental in the setting of chronic neuroinflammation, downregulating melatonin may be beneficial in activating innate immune response in the context of tumor-mediated immune suppression.

## Conclusion

In GBM, TAMs are increasingly recognized to play a critical role in shaping the tumor microenvironment that could affect prognosis and response to therapy. Understanding the plasticity and functional heterogeneity of TAMs is crucial in developing therapeutics targeting TAMs. Mitochondrial dysfunctional in GBM is a well-known phenomenon, but how it affects TAMs functionally and thereby the immune microenvironment has been largely unexplored. A growing number of studies, in other disease and cancer models, suggest that mtDNA release is a significant byproduct of mitochondrial dysfunction and that mtDNA can potently activate STING-dependent type I IFN production. Whether this dynamic holds true in the unique GBM microenvironment with an abundance of blood-derived macrophages and resident microglia has not been explored. Understanding this cross talk between GBM mitochondrial dysfunction and phenotypical subsets of TAMs may be crucial in developing novel therapeutic strategies against GBM.

## Author Contributions

RL provided ideas for the project and wrote the initial draft. WH completed final revision. All authors read and approved the final manuscript.

## Conflict of Interest

The authors declare that the research was conducted in the absence of any commercial or financial relationships that could be construed as a potential conflict of interest.
